# Feline Otitis Externa Caused by Methicillin-Resistant *Staphylococcus aureus* with Mixed Hemolytic Phenotype and Overview of Possible Genetic Backgrounds

**DOI:** 10.3390/antibiotics10050599

**Published:** 2021-05-18

**Authors:** Jana Avberšek, Bojan Papić, Darja Kušar, Vladimira Erjavec, Katja Seme, Majda Golob, Irena Zdovc

**Affiliations:** 1 Institute of Microbiology and Parasitology, Veterinary Faculty, University of Ljubljana, Gerbičeva 60, SI-1000 Ljubljana, Slovenia; jana.avbersek@vf.uni-lj.si (J.A.); bojan.papic@vf.uni-lj.si (B.P.); darja.kusar@vf.uni-lj.si (D.K.); majda.golob@vf.uni-lj.si (M.G.); 2 Small Animal Clinic, Veterinary Faculty, University of Ljubljana, Cesta v Mestni log 47, SI-1000 Ljubljana, Slovenia; Vladimira.Erjavec@vf.uni-lj.si; 3 Institute of Microbiology and Immunology, Faculty of Medicine, University of Ljubljana, Zaloška 4, SI-1000 Ljubljana, Slovenia; katja.seme@mf.uni-lj.si

**Keywords:** methicillin-resistant *Staphylococcus aureus* (MRSA), otitis externa, cat, whole-genome sequencing (WGS), mixed hemolysis

## Abstract

Methicillin-resistant *Staphylococcus aureus* (MRSA) is an important cause of nosocomial infections in humans, but its importance in small animal practice is increasing. Here, we present a case of feline otitis externa (OE) caused by MRSA; both hemolytic and nonhemolytic variants with a stable phenotype were recovered from the external auditory canal after infection was detected by routine otoscopy. One isolate per variant underwent antimicrobial susceptibility testing (AST) by broth microdilution method, conventional *spa* typing and whole-genome sequencing (WGS). The results showed that both variants were genetically related and were of sequence type (ST) 1327, SCC*mec* type IV and *spa* type t005. AST and WGS showed that both isolates were resistant to β-lactams and sensitive to all tested non-β-lactam antibiotics. Both isolates were *pvl*-negative, but encoded several other virulence genes (*aur*, *hlgABC*, *sak*, *scn*, *seg*, *sei*, *sem*, *sen*, *seo* and *seu*). Genetic background of the mixed hemolytic phenotype was not identified; no differences in the *agr* locus or other regulatory regions were detected. Three single-nucleotide polymorphisms were identified but could not be associated with hemolysis. This well-documented case of MRSA infection in companion animals adds to the reports of MRSA infections with a mixed hemolytic phenotype.

## 1. Introduction

Methicillin-resistant *Staphylococcus aureus* (MRSA) is a significant global healthcare problem in both human and veterinary medicine. Recent data suggest an increase in MRSA infections, particularly in companion animals, which poses a risk for nosocomial spread in veterinary hospitals [1,2,3,4]. In companion animals, MRSA-associated wound infections have been frequently described [2,4]. Moreover, companion animals of MRSA-infected human patients were reported as positive in approximately 10% of households, suggesting they can be a source of (re)infection for humans [5,6]. On the other hand, owners of MRSA-infected companion animals represent a source of animal or human (re)infection but are more likely to be colonized than infected [7]. Probable transmission of *S. aureus* (including MRSA) between companion animals and humans (zoonosis) and vice versa (anthroponosis) is frequently reported [4,5,6,7,8,9]. Although MRSA is not a common colonizer of cats, cases of MRSA infection have been documented in cats and their owners [10,11,12].

The ability of *S. aureus* to cause disease is associated with a large number of virulence factors that are highly dependent on the site of infection; *S. aureus* secretes numerous exotoxins such as α-hemolysin (encoded by *hla*), bi-component leukocidins (e.g., Panton Valentine leukocidin, encoded by *pvl*, and γ-hemolysin, encoded by *hlg*), β-hemolysin (encoded by *hlb*) and phenol-soluble modulins (PSMs) [13,14]. In *S. aureus*, the expression of secreted (e.g., hemolysins) and cell-associated proteins involved in the virulence is coordinated globally by a complex quorum-sensing (QS) operon, called the accessory gene regulator (*agr*). It is controlled by two promoters: P2 directs the synthesis of RNAII (encoding genes *agrA*–*D*, involved in QS signal transduction) and P3 the synthesis of RNAIII [15,16]. The latter encodes δ-hemolysin (encoded by *hld*), a membrane-damaging PSM [13]; however, RNAIII itself, in combination with various cofactors, acts as a global regulator of exoprotein genes at the level of transcription and translation [15,16,17]. A high degree of heterogeneity in *S. aureus* isolates results from the presence/absence of toxin genes, disruption of gene loci and variations in gene expression levels [13].

Several studies have reported mixed (heterogeneous) *S. aureus* infections in humans where both hemolytic and nonhemolytic variants were obtained, resulting from mutations in the *agr* locus or elsewhere [18,19]. Strains with low or no Agr activity have been reported among *S. aureus* colonizing healthy individuals, hospital-acquired (HA) MRSA and also MRSA strains of various animal origins [18,20,21].

Studies of MRSA infections with a mixed hemolytic phenotype in animals are currently lacking. The aim of the present study was to characterize two MRSA variants, one with a hemolytic and one with a nonhemolytic phenotype, originating from a cat with acute otitis externa (OE) diagnosed during routine otoscopy. To this aim, the isolates underwent antimicrobial susceptibility testing, *spa* typing and whole-genome sequencing. In addition, an overview of possible genetic backgrounds influencing the mixed hemolytic phenotype is provided.

## 2. Results

### 2.1. Case Report

A 5-year-old, neutered male, short-haired domestic cat weighing 4.0 kg was presented to the Small Animal Clinic of the Veterinary Faculty in Ljubljana, Slovenia. Prior to the visit to the clinic, the cat owner entered a nursing home, after which the cat was adopted by his daughter. The first owner had been hospitalized before and after entering the nursing home. The new owner brought the animal to the clinic for routine dental treatment. Since the cat was anesthetized, clinical examination was also performed because brown stains were noticed on the cat’s bedding. Complete blood count and biochemistry revealed no abnormalities, the FIV (feline immunodeficiency virus) and FeLV (feline leukemia virus) tests were negative. After scaling and polishing the teeth, the upper left fourth premolar was surgically removed due to advanced periodontal disease. While rhinoscopy revealed no abnormalities, otoscopy showed that the left ear canal was filled with yellow, creamy discharge. The right ear canal had dry brown material and the tympanic membrane was ruptured. Cytologic evaluation of the exudate from the left ear canal revealed the presence of neutrophils and multiple coccoid bacteria. The cat underwent systemic antimicrobial therapy with amoxicillin/clavulanic acid (Synulox; Pfizer, Rome, Italy; 50 mg, 1.5 tablets twice a day). Control otoscopy was performed ten days later and antimicrobial therapy was prolonged because the response to the treatment was poor and the ear canals were still inflamed. One week later, the cat was presented again with a complaint of odor from the left ear. Otoscopy of the right ear canal revealed no abnormalities, while the left ear canal showed a yellow, creamy discharge. A swab was taken from the left ear canal for bacteriological and mycological examination. In addition, swabs were taken from the right ear canal, nasal cavity, pharynx, gingiva and both conjunctivae for bacteriological and mycological examination. On the same day, antimicrobial therapy was changed to enrofloxacin (Enroxil; Krka, Novo mesto, Slovenia; 15 mg, 1.5 tablets once a day) and continued for two weeks. Antibiotics were discontinued after a negative bacteriological result of the left ear canal swab was obtained.

After MRSA identification in the examined cat, swabs of the left and right ear canals were obtained from two other cats from the same household that had been in contact with the MRSA-positive cat; pharyngeal and gingival swabs were also obtained from one of the two cats. Nasal, pharyngeal and gingival swabs were also taken from the new cat owner and her father for MRSA detection.

### 2.2. Bacteriological Examination and Antimicrobial Susceptibility Testing (AST)

Only samples from the left ear canal of the OE-affected cat were positive. Abundant bacterial growth was observed on the blood agar plates dominated by hemolytic and nonhemolytic colonies. Both hemolytic and nonhemolytic phenotypes were stable even after ten consecutive passages on blood agar. Both types of colonies were coagulase-positive, agglutinated by the Monostaph Plus and identified as *S. aureus* using API Staph. The third, less dominant, dispersed single colonies showed a wide incomplete hemolysis and were identified as *Staphylococcus pseudintermedius* using the same protocol as for S. aureus. A pure culture of lipophilic yeast *Malassezia pachydermatis* was observed on the Sabouraud dextrose agar plates after two days of incubation at 37 °C. Samples obtained from the right ear canal, nasal cavities, pharynx, gingiva and both conjunctivae of the OE-affected cat were bacteriologically negative, with only a few colonies of the normal skin microbiota present. In addition, the samples obtained from two other cats and human samples were also MRSA-negative.

*S. pseudintermedius* was susceptible to all the tested antimicrobials, whereas both MRSA isolates were resistant to penicillin, ampicillin, oxacillin and cefoxitin (Table 1).

### 2.3. Molecular Characterization and Whole-Genome Sequencing

Results of conventional PCR and WGS (Table 2) confirmed that both isolates are MRSA. Phenotypic and genotypic AST results were concordant. Both assembled genomes passed the quality check with BioNumerics and showed (*i*) *N*_50_ of 101,126 kb (SA36) and 152,361 kb (SA37), (*ii*) total assembly length of 2.8 Mb (SA36 and SA37) and (*iii*) number of contigs of 63 (SA36) and 54 (SA37). Both isolates harbored virulence genes encoding γ-hemolysin (*hlgABC*), aureolysin (*aur*), several staphylococcal enterotoxins (*seg*, *sei*, *sem*, *sen*, *seo* and *seu*), and two immune evasion cluster (IEC) genes encoding staphylokinase (*sak*) and staphylococcal complement inhibitor (*scn*). None of the isolates harbored *pvl* gene.

The whole-genome multilocus sequence typing (wgMLST) analysis revealed that the isolates differed in a single allele in the *fnbA* locus (encoding fibronectin-binding protein A); however, this locus had multiple allele hits due to a large number of heterozygous single nucleotide polymorphisms (SNPs) and was thus considered unreliable. Genome-wide single nucleotide (SNP) typing revealed three SNPs that differentiated the studied MRSA isolates with mixed hemolytic phenotype (Table 3). In addition to two SNPs in the noncoding region, a missense SNP mutation was identified in *cycA* gene encoding serine/alanine/glycine permease, resulting in amino acid substitution A331S in the isolate SA37. Sequence analysis revealed no differences between hemolytic and nonhemolytic MRSA in the hemolysin genes (*hla*, *hlb*, *hld* and *hlgC*), regulator genes (*sigB*, *saeR*, *ccpA*, *sarART* and *agrR*) or genes present in the *agr* locus (*agrA*–*D* and RNAIII gene); therefore, the genetic background leading to the nonhemolytic phenotype was not identified.

## 3. Discussion

In the present study, two MRSA variants were identified in the infected feline ear canal, one hemolytic and one nonhemolytic, which did not differ in any other studied phenotypic or genotypic traits. Despite their genomic analysis, the cause of mixed OE infection could not be determined. *S. pseudintermedius* and *M. pachydermatis* were also isolated from the ear canal, but were not considered to be the cause of OE because the disease did not subside after the treatment with amoxicillin/clavulanic acid, which should be effective against *S. pseudintermedius*. In addition, OE subsided after the treatment with enrofloxacin and without antifungal agents. We could not reliably confirm whether both MRSA variants were responsible for the infection, but infections with mixed hemolytic variants are commonly reported in humans, especially in association with the disrupted QS regulatory network of *S. aureus*.

In *S. aureus*, 16 two-component regulatory systems have been described [22]. Among them, *agr* was one of the first and best studied [15,16,23], but other regulatory loci have also been described in *S. aureus* such as *sar*, *sigB*, *sae* and *arl*, as well as several proteins with homology to SarA such as Rot (repressor of toxins), which act as cross-talking global regulators of virulence genes and have both positive and negative effects on gene expression [17]. Due to RNAIII of the *agr* operon functioning as a specific regulator of exoprotein gene expression in *S. aureus* and possibly requiring a conformational change to activate its function [15], deletions or other mutations affecting the secondary or tertiary structure of RNAIII could affect the hemolytic phenotype of *S. aureus*. RNAIII regulates gene transcription via accessory protein factors, possibly by directly interacting with and antagonizing transcription activators [15]. It has also been speculated that RNAIII interacts directly with regulatory sequences in target DNA [15]. On the other hand, RNAIII may regulate translation, which has been reported for α-hemolysin [15]. The insertion of transposon Tn*551*, which eliminates the production of α-hemolysin without affecting the structural gene, has also been described to affect the regulation of virulence phenotype in *S. aureus* [24]. Such a pleiotropic mutation by transposon insertion leading to decreased production of hemolysins has also been described for the regulatory loci *sae* and *sarA*, and SarA is also known to bind to DNA located between the two *agr* promoters [25].

Due to the complexity of virulence gene regulation in *S. aureus*, it is not always possible to identify the reason for the altered phenotype, as observed herein. It has been previously reported that even large differences in the virulence of *S. aureus* strains can result from relatively small differences in their genomes [26], such as SNPs leading to reduced promoter-binding affinity of the mutated regulatory proteins [27] or mobile genetic elements [28]. Previous studies examining the within-host diversity of *S. aureus* using WGS revealed a fairly high genetic diversity of *S. aureus*, reaching up to 40 SNPs in infecting populations [29], which highlights the advantage of selecting more than one isolate per sample in epidemiological studies.

In the present study, *fnbA* was the only locus with differences in consensus allele calls when hemolytic and nonhemolytic MRSA variants were compared using wgMLST, but this difference could not be reliably confirmed due to the presence of heterozygous SNPs. Nevertheless, FnbA and FnbB are large adhesive proteins important for the virulence of *S. aureus* and are under direct regulation of SarA; they promote bacterial adherence to biotic or abiotic surfaces (cell-to-surface interactions) and are involved in evasion of host immune responses [30,31]. Since SarA also binds to the hemolysis-affecting *agr* regulon [25], a high complexity of *S. aureus* regulatory networks can be inferred. A single missense mutation differentiating the studied MRSA isolates was identified in *cycA* gene. The disruption of this gene has been shown to increase the susceptibility to β-lactams [32]; however, its role in hemolysis is currently unknown. None of the identified intergenic mutations were located in the *agr* locus. The role of the identified SNPs in the mixed hemolytic phenotype remains to be elucidated in future experimental studies.

Infections with mixed hemolytic and nonhemolytic subpopulations of *S. aureus* isolates have been shown to result from mutations in *agr* or other loci during infection rather than subculturing after isolation [18], although revertants have been reported for approximately 10% of human clinical cases [33] and frequent spontaneous *agr* mutations have been observed in the laboratory due to a high metabolic burden entailed by the *agr* autoactivation circuit [16]. In the present study, the nonhemolytic MRSA strain did not revert back to hemolytic after several serial passages on the growth medium. For human clinical cases, invasive *S. aureus* infections with dysfunctional *agr* have been also associated with unfavorable clinical outcomes, depending on the site of infection and resistance to oxacillin [34]. In the present case, the OE infection in the cat was successfully resolved by enrofloxacin. It was reported that the presence of *agr*-defective strains is strongly associated with hospitalization or prior use of antibiotics such as fluoroquinolones or β-lactams, and also that community-acquired (CA) MRSA strains can trade Agr activity for methicillin resistance upon exposure to β-lactams [19,35]. In the present study, unfortunately, no MRSA isolate could be obtained from the cat owners to allow comparison and to determine the source of MRSA.

The obtained MRSA strains exhibited *agr* type I. The *agr* groups have been reported to be biologically and clinically significant [16]. Despite the commonly described association between Agr activity and hemolysis, it has also been suggested that the absence of hemolysis may not be sufficiently sensitive to determine Agr activity and *agr*-defective strains should undergo genome sequencing [19]. However, in the present study, despite performing WGS-based comparisons, we did not detect any differences between the two MRSA strains. Since we detected the presence of ΦSa3, which encodes *sak* and *scn*, it is also possible that prophages are involved in the mixed hemolytic phenotype via lysogenic conversion [36]; namely, ΦSa3It is a β-hemolysin-converting prophage that is present in most human *S. aureus* clones and generates non-functional *hlb* variants [37].

The rates of MRSA carriage in healthy cats and dogs remain poorly defined but are reported to be low (2–3%) [38]. Numerous reports, as well as epidemiological data, suggest nosocomial spread of MRSA within small and large animal practices [1,39,40]. The potential for anthroponotic transmission and the creation of animal reservoirs for human reinfection are major public health concerns. There is evidence of bidirectional MRSA transmission between humans and their pets [5,39]. A case study conducted by Vitale et al. [11] described the first case of isolation of MRSA USA300, a CA-MRSA clone from a household pet. Indistinguishable MRSA USA300 was isolated from the skin lesions of a cat and the cat’s owner who reported skin abscesses and pneumonia three months earlier. It was therefore likely that MRSA was transmitted between the cat and the owner, but it could not be definitively confirmed whether this was a case of zoonotic or anthroponotic transmission. In this study, we were unable to confirm the transmission of MRSA from the owner to the cat because both the first and the second cat owners were MRSA-negative at the time of sampling. Interestingly, the second owner frequently fed the cat leftover food from a nursing home. A retrograde investigation of MRSA isolates from this nursing home was performed in the MRSA strain collection of Medical Faculty, Institute of Microbiology and Immunology. Two isolates were identified, but they had a different resistance profile than the isolates obtained from the cat (data not shown).

The distinction between HA- and CA-MRSA has become significantly blurred since CA strains are increasingly observed in the nosocomial infections and HA strains in community settings [41]. The coexistence of HA and CA strains is being maintained by high rates of cycling individuals between the healthcare settings and the community, and supported by a large heterogeneity of the human population [42]. Traditionally, the distinction between CA- and HA-MRSA has been based on SCC*mec* typing, the presence of *pvl* gene and susceptibility to non-β-lactam antibiotics [43]. However, in this study, both MRSA isolates were *pvl*-negative, belonged to the CC22 lineage and harbored SCC*mec* type IV. CC22 lineage is a successful HA lineage, but is also commonly reported in pets and their owners [2,12,44]. In a previous study, MRSA from pets was also *pvl*-negative and belonged to the HA-MRSA clone ST22–SCC*mec* IV, but showed *spa* type t032 [45]. Since MRSA isolates from animal patients can reflect typical human HA lineages such as CC22, including MLST and *spa* types similar to those of human origin, nasal carriage among veterinarians or pet owners should not be neglected [46]. Moreover, WGS-based phylogeny of human, feline and canine MRSA isolates belonging to ST22 showed that isolates from companion animal isolates clustered within the epidemic MRSA-15 (EMRSA-15) pandemic clade, and in a way that human source could be suggested for animal infections [47]. Furthermore, as in human hospitals, veterinary nosocomial transmission of MRSA has been confirmed [47]. Not only can successful MRSA lineages can be shared and readily exchanged between humans and companion animals, but they could also cause infections without prior host adaptations, suggested as an ‘extended-spectrum genotype’ [47].

## 4. Materials and Methods

### 4.1. Bacteriological Examination

For bacteriological examination, samples were streaked onto nutrient agar (Oxoid, UK) supplemented with 5% sheep blood, Drigalski agar and Sabouraud dextrose agar (Oxoid, UK) supplemented with chloramphenicol (100 mg/L). Plates were incubated at 37 °C for 48 h. The obtained cultures were tested for coagulase (Biolife Italiana, Milano, Italy) and underwent the Monostaph Plus (Bionor Laboratories AS, Skien, Norway) rapid latex agglutination test to distinguish *S. aureus* from other *Staphylococcus* species; the test detects both MRSA and methicillin-susceptible *S. aureus* (MSSA). Biochemical characteristics were evaluated using the commercial API Staph kit (BioMérieux, Marcy-l’Étoile, France) according to the manufacturer’s instructions. Prior to WGS, all isolates were confirmed using the matrix-assisted laser desorption/ionization time-of-flight (MALDI-TOF) mass spectrometry (Microflex LT system; Bruker Daltonics, Bremen, Germany) according to the manufacturer’s instructions.

### 4.2. Antimicrobial Susceptibility Testing

Three isolates obtained from the OE-affected cat (hemolytic and nonhemolytic MRSA and *S. pseudintermedius*; all from the left ear canal) underwent AST. AST was performed on commercial 96-well broth microdilution plates for Gram-positive bacteria, Sensititre Gram Positive GPALL1F plates (TREK Diagnostic Systems; Thermo Fisher Scientific, Oxford, UK) according to the manufacturer’s instructions. The antimicrobials tested were ampicillin, chloramphenicol, ciprofloxacin, clindamycin, daptomycin, erythromycin, gentamycin, levofloxacin, linezolid, moxifloxacin, nitrofurantoin, oxacillin, penicillin, quinupristin/dalfopristin, rifampin, streptomycin, tetracycline, tigecycline, trimethoprim/sulfamethoxazole, vancomycin and cefoxitin indicator. The results of the minimum inhibitory concentration (MIC) were evaluated according to the Clinical and Laboratory Standards Institute criteria for *Staphylococcus* spp. or *S. aureus* where available [48]. The following control strains were included: *S. aureus* ATCC 29213 as MRSA-negative control and the in-house *S. aureus* pig isolate (ST398) as MRSA-positive control.

### 4.3. Molecular Characterization

The obtained isolates were subjected to identification by multiplex PCR assay. The following primer sets were used: (*i*) *mecA*-1 (GGG ATC ATA GCG TCA TTA TTC) and *mecA*-2 (AAC GAT TGT GAC ACG ATA GCC), which amplify a 527-bp fragment of the *mecA* gene; (*ii*) *nuc-*1 (TCA GCA AAT GCA TCA CAA ACA G) and *nuc*-2 (CGT AAA TGC ACT TGC TTC AGG), which amplify a 255-bp fragment of the *S. aureus* nuclease (*nuc*) gene; and (*iii*) 16S-1 (GTG CCA GCA GCC GCG GTA A) and 16S-2 (AGA CCC GGG AAC GTA TTC AC), which amplify a 886-bp fragment of the *Staphylococcus* sp. 16S rRNA gene [49]. DNA was extracted from bacterial cultures using a simple cell lysis (boiling at 95 °C for 15 min, centrifugation at 14,000× *g* for 2 min) and the supernatant was used as the template for PCR. A 25-µL reaction mixture contained 12.5 µL of 2× Multiplex PCR Kit (Qiagen, Hilden, Germany), 0.2 μM of each primer (Invitrogen, Waltham, MA, USA), 1 µL of template DNA and PCR-grade water to a final volume of 25 μL. Amplification was performed according to the following protocol: initial denaturation at 95 °C for 15 min, 30 cycles of denaturation at 94 °C for 30 s, annealing at 55 °C for 30 s and extension at 72 °C for 1 min, and final extension at 72 °C for 10 min. PCR amplicons were separated by electrophoresis in 1.5% agarose gels, stained with ethidium bromide and visualized under UV light.

For *spa* typing, the *spa* gene was amplified as previously described [50]. Amplicons were inspected by electrophoresis in 1.5% agarose gels, stained with ethidium bromide and visualized under UV light, followed by Sanger sequencing (SEQme, Dobříš, Czech Republic). *Spa* types were assigned using the *spa*-typing plugin of the BioNumerics v.7.6.3 software (bioMérieux, Applied Maths NV, Sint-Martens-Latem, Belgium).

### 4.4. Whole-Genome Sequencing

Both the hemolytic and nonhemolytic MRSA isolates underwent WGS and comprehensive WGS-based characterization. For WGS, genomic DNA was extracted using the DNA Blood & Tissue Kit (Qiagen, Hilden, Germany) and DNA libraries were prepared using the Illumina TruSeq DNA Nano Library Prep Kit (Illumina, San Diego, CA, USA). Sequencing was performed on the NextSeq500 System using the 2 × 150 bp chemistry (Illumina, USA) to a minimum coverage of 170 ×. Sequencing data of the nonhemolytic MRSA isolate SA36 and hemolytic MRSA isolate SA37 were submitted to the NCBI Sequence Read Archive (SRA) database under run accession numbers SRR7647329 and SRR7647328, respectively.

Reads were assembled using SPAdes integrated into the BioNumerics v7.6.3 software (bioMérieux, Applied Maths NV, Martens-Latem, Belgium). SCC*mec* type was identified using SCC*mec*Finder v1.2 [51] by applying the default parameters. Antimicrobial resistance genes were identified using ResFinder v3.2 [52] by applying the default parameters. *In silico* MLST was performed using the “Sequence query” tool implemented in the *S. aureus* BIGSdb website (https://pubmlst.org/saureus/, accessed on 2 March 2021). wgMLST based on the *S. aureus* wgMLST scheme consisting of 3897 loci was performed using BioNumerics; default settings were used for both assembly-free and assembly-based allele calling.

Genome-wide SNP typing was performed in BioNumerics by applying the default settings for read mapping against the ST22 reference genome HO 5096 0412 (NCBI accession number HE681097.1) using Bowtie (minimum total coverage of 3, minimum forward and reverse coverage both of 1). After mapping, SNPs were called by applying the strict SNP filtering template (minimum inter-SNP distance of 12 bp, minimum total coverage of 5, minimum forward coverage and reverse coverage both of 1). Non-informative SNP positions and positions with gaps, ambiguous or unreliable bases were removed from the analysis.

Sequence analysis of the *S. aureus* hemolysin genes *hla*, *hlb*, *hlgC*, *hld*, the regulator genes *sigB*, *saeR*, *ccpA*, *sarART*, *arlRS*, *agrR*, and the *agr* locus was performed using Geneious v11.1.5 (Biomatters, Auckland, New Zealand).

## 5. Conclusions

Here, we present a well-documented case of MRSA infection in companion animals with mixed hemolytic phenotype. One hemolytic and one nonhemolytic MRSA variant were identified in the infected feline ear canal, both characterized as *spa* type t005, ST1327, SCC*mec* IV and *agr* type I. Their antimicrobial resistance profiles were identical and no differences were observed in the hemolysin genes, regulatory genes and *agr* locus.

## Figures and Tables

**Table 1:** Results of antimicrobial susceptibility testing for three *Staphylococcus* isolates.

Antimicrobial	Isolate					
	Hemolytic MRSA Isolate SA37		Nonhemolytic MRSA Isolate SA36		*S. pseudintermedius*	
	MIC (µg/mL)	S/R	MIC (µg/mL)	S/R	MIC (µg/mL)	S/R
**Ampicillin**	**8**	**R**	**4**	**R**	≤0.125	S
**Cefoxitin**	**>6**	**R**	**>6**	**R**	≤6	/
Chloramphenicol	8	S	8	S	8	S
Ciprofloxacin	≤1	S	≤1	S	≤1	S
Clindamycin	≤0.5	S	≤0.5	S	≤0.5	S
Daptomycin	≤0.5	S	≤0.5	S	≤0.5	S
Erythromycin	≤0.25	S	≤0.25	S	0.5	S
Gentamycin	≤2	S	≤2	S	≤2	S
Levofloxacin	≤0.25	S	≤0.25	S	≤0.25	S
Linezolid	≤1	S	≤1	S	≤1	S
Moxifloxacin	≤0.25	S	≤0.25	S	≤0.25	S
Nitrofurantoin	≤32	S	≤32	S	≤32	S
**Oxacillin**	**>4**	**R**	**>4**	**R**	≤0.25	S
**Penicillin**	**>8**	**R**	**>8**	**R**	≤0.06	S
Quinupristin/dalfopristin	≤0.5	S	≤0.5	S	≤0.5	S
Rifampin	≤0.5	S	≤0.5	S	≤0.5	S
Streptomycin	≤1000	S	≤1000	S	≤1000	S
Tetracycline	≤2	S	≤2	S	≤2	S
Tigecycline	0.25	S	0.125	S	≤0.06	S
Trimethoprim/sulfamethoxazole	≤0.5	S	≤0.5	S	≤0.5	S
Vancomycin	0.5	S	0.5	S	0.5	S

S, susceptible; R, resistant. All MIC values shown in bold were interpreted as resistant.

**Table 2:** Genetic characteristics of the hemolytic MRSA isolate SA37 and the nonhemolytic MRSA isolate SA36. Note that only the genes that are present are reported.

	Gene or Genotype (Isolates SA36 and SA37)
mPCR	16S rRNA, *nuc*, *mecA*
*spa* type	t005
MLST ST (CC)	ST1327 (CC22)
SCC*mec* type	IV
*agr* type	I
Resistance genes	*blaZ*, *mecA* (conferring resistance to β-lactams)
Virulence genes	*aur*, *hlgABC*, *sak*, *scn*, *seg*, *sei*, *sem*, *sen*, *seo*, *seu*

mPCR, multiplex PCR; CC, clonal complex; ST, sequence type.

**Table 3:** SNPs differentiating the hemolytic MRSA isolate SA37 and the nonhemolytic MRSA isolate SA36. All SNP positions are relative to the reference genome HE681097.1.

Position	CDS/Intergenic	Gene	Nucleotide: Isolate SA36	Nucleotide: Isolate SA37
1491402	Intergenic		G	T
1765477	CDS	*cycA*	G	T
2329625	Intergenic		G	A

CDS, coding sequence.

## Data Availability

The data presented in this study are available in NCBI Sequence Read Archive (SRA) database under the BioProject accession number PRJNA484707.

## References

[1] Walther B., Wieler L.H., Friedrich A.W., Kohn B., Brunnberg L., Lübke-Becker A. (2009). *Staphylococcus aureus* and MRSA colo-nization rates among personnel and dogs in a small animal hospital: Association with nosocomial infections. Berl. Munch. Ti-erarztl. Wochenschr..

[2] Vincze S., Stamm I., Kopp P.A., Hermes J., Adlhoch C., Semmler T., Wieler L.H., Lübke-Becker A., Walther B. (2014). Alarming proportions of methicillin-resistant *Staphylococcus aureus* (MRSA) in wound samples from companion animals, Germany 2010–2012. PLoS ONE.

[3] Loncaric I., Lepuschitz S., Ruppitsch W., Trstan A., Andreadis T., Bouchlis N., Marbach H., Schauer B., Szostak M.P., Feßler A.T. (2019). Increased genetic diversity of methicillin-resistant *Staphylococcus aureus* (MRSA) isolated from companion animals. Vet. Microbiol..

[4] Penna B., Silva M.B., Soares A.E.R., Vasconcelos A.T.R., Ramundo M.S., Ferreira F.A., Silva-Carvalho M.C., de Sousa V.S., Rabello R.F., Bandeira P.T. (2021). Comparative genomics of MRSA strains from human and canine origins reveals similar virulence gene repertoire. Sci. Rep..

[5] Ferreira J.P., Anderson K.L., Correa M.T., Lyman R., Ruffin F., Reller L.B., Fowler V.G. (2011). Transmission of MRSA between companion animals and infected human patients presenting to outpatient medical care facilities. PLoS ONE.

[6] Morris D.O., Lautenbach E., Zaoutis T., Leckerman K., Edelstein P.H., Rankin S.C. (2012). Potential for pet animals to harbour methicillin-resistant *Staphylococcus aureus* when residing with human MRSA patients. Zoonoses Public Health.

[7] Morgan M. (2008). Methicillin-resistant *Staphylococcus aureus* and animals: Zoonosis or humanosis?. J. Antimicrob. Chemother..

[8] Asanin J., Misic D., Aksentijevic K., Tambur Z., Rakonjac B., Kovacevic I., Spergser J., Loncaric I. (2019). Genetic profiling and comparison of human and animal methicillin-resistant *Staphylococcus aureus* (MRSA) isolates from Serbia. Antibiotics.

[9] Hogan P.G., Mork R.L., Boyle M.G., Muenks C.E., Morelli J.J., Thompson R.M., Sullivan M.L., Gehlert S.J., Merlo J.R., McKenzie M.G. (2019). Interplay of personal, pet, and environmental colonization in households affected by community-associated methicillin-resistant *Staphylococcus aureus*. J. Infect..

[10] Scott G., Thomson R., Malone-Lee J., Ridgway G. (1988). Cross-infection between animals and man: Possible feline transmission of *Staphylococcus aureus* infection in humans?. J. Hosp. Infect..

[11] Vitale C.B., Gross T., Weese J.S. (2006). Methicillin-resistant *Staphylococcus aureus* in cat and owner. Emerg. Infect. Dis..

[12] Wipf J., Perreten V. (2016). Methicillin-resistant *Staphylococcus aureus* isolated from dogs and cats in Switzerland. Schweiz. Arch. Tierheilkd..

[13] Evandenesch F., Elina G., Ehenry T. (2012). *Staphylococcus aureus* hemolysins, bi-component leukocidins, and cytolytic peptides: A redundant arsenal of membrane-damaging virulence factors?. Front. Cell. Infect. Microbiol..

[14] Pontieri E., Savini V. (2018). The staphylococcal hemolysins. Pet-to Man Travelling Staphylococci. A World in Progress.

[15] Novick R., Ross H., Projan S., Kornblum J., Kreiswirth B., Moghazeh S. (1993). Synthesis of staphylococcal virulence factors is controlled by a regulatory RNA molecule. EMBO J..

[16] Novick R.P., Geisinger E. (2008). Quorum sensing in staphylococci. Annu. Rev. Genet..

[17] Saìd-Salim B., Dunman P.M., McAleese F.M., Macapagal D., Murphy E., McNamara P.J., Arvidson S., Foster T.J., Projan S.J., Kreiswirth B.N. (2003). Global regulation of *Staphylococcus aureus* genes by Rot. J. Bacteriol..

[18] Traber K.E., Lee E., Benson S., Corrigan R., Cantera M., Shopsin B., Novick R.P. (2008). *agr* function in clinical *Staphylococcus aureus* isolates. Microbiol..

[19] Painter K.L., Krishna A., Wigneshweraraj S., Edwards A.M. (2014). What role does the quorum-sensing accessory gene regulator system play during *Staphylococcus aureus* bacteremia?. Trends Microbiol..

[20] Shopsin B., Drlica-Wagner A., Mathema B., Adhikari R.P., Kreiswirth B.N., Novick R.P. (2008). Prevalence of *agr* dysfunction among colonizing *Staphylococcus aureus* strains. J. Infect. Dis..

[21] Huber C., Stamm I., Ziebuhr W., Marincola G., Bischoff M., Strommenger B., Jaschkowitz G., Marciniak T., Cuny C., Witte W. (2020). Silence as a way of niche adaptation: *mecC*-MRSA with variations in the accessory gene regulator (*agr*) functionality express kaleidoscopic phenotypes. Sci. Rep..

[22] Cheung A.L., Zhang G. (2002). Global regulation of virulence determinants in *Staphylococcus aureus* by the SarA protein family. Front. Biosci.

[23] Recsei P., Kreiswirth B., O’Reilly M., Schlievert P., Gruss A., Novick R.P. (1986). Regulation of exoprotein gene expression in *Staphylococcus aureus* by *agr*. Mol. Genet. Genom..

[24] Mallonee D.H., A Glatz B., A Pattee P. (1982). Chromosomal mapping of a gene affecting enterotoxin A production in *Staphylococcus aureus*. Appl. Environ. Microbiol..

[25] Giraudo A.T., Calzolari A., Cataldi A.A., Bogni C., Nagel R. (1999). The *sae* locus of *Staphylococcus aureus* encodes a two-component regulatory system. FEMS Microbiol. Lett..

[26] Kennedy A.D., Otto M., Braughton K.R., Whitney A.R., Chen L., Mathema B., Mediavilla J.R., Byrne K.A., Parkins L.D., Tenover F.C. (2008). Epidemic community-associated methicillin-resistant *Staphylococcus aureus*: Recent clonal expansion and diversification. Proc. Natl. Acad. Sci. USA.

[27] Sause W.E., Copin R., O’Malley A., Chan R., Morrow B.J., Buckley P.T., Fernandez J., Lynch A.S., Shopsin B., Torres V.J. (2017). *Staphylococcus aureus* strain Newman D2C contains mutations in major regulatory pathways that cripple its pathogenesis. J. Bacteriol..

[28] McClure J.-A.M., Lakhundi S., Kashif A., Conly J.M., Zhang K. (2018). Genomic comparison of highly virulent, moderately virulent, and avirulent strains from a genetically closely-related MRSA ST239 sub-lineage provides insights into pathogenesis. Front. Microbiol..

[29] Fitzgerald J.R., Holden M.T. (2016). Genomics of natural populations of *Staphylococcus aureus*. Annu. Rev. Microbiol..

[30] Shanks R.M.Q., Meehl M.A., Brothers K.M., Martinez R.M., Donegan N.P., Graber M.L., Cheung A.L., O’Toole G.A. (2008). Genetic Evidence for an alternative citrate-dependent biofilm formation pathway in *Staphylococcus aureus* that is dependent on fibronectin binding proteins and the GraRS two-component regulatory system. Infect. Immun..

[31] Stutz K., Stephan R., Tasara T. (2010). SpA, ClfA, and FnbA genetic variations lead to Staphaurex test-negative phenotypes in bovine mastitis *Staphylococcus aureus* isolates. J. Clin. Microbiol..

[32] Gallagher L.A., Shears R.K., Fingleton C., Alvarez L., Waters E.M., Clarke J., Bricio-Moreno L., Campbell C., Yadav A.K., Razvi F. (2020). Impaired alanine transport or exposure to D-cycloserine increases the susceptibility of MRSA to β-lactam antibiotics. J. Infect. Dis..

[33] Gor V., Hoshi M., Takemura A.J., Higashide M., Romero V.M., Ohniwa R.L., Morikawa K. (2021). Virulence reversion in *Staphylococcus aureus*. Proceedings.

[34] Lee S.O., Lee S., Lee J.E., Song K.-H., Kang C.K., Wi Y.M., San-Juan R., López-Cortés L.E., Lacoma A., Prat C. (2020). Dysfunctional accessory gene regulator (*agr*) as a prognostic factor in invasive *Staphylococcus aureus* infection: A systematic review and meta-analysis. Sci. Rep..

[35] Paulander W., Varming A.N., Bæk K.T., Haaber J., Frees D., Ingmer H. (2012). Antibiotic-mediated selection of quorum-sensing-negative *Staphylococcus aureus*. mBio.

[36] Xia G., Wolz C. (2014). Phages of *Staphylococcus aureus* and their impact on host evolution. Infect. Genet. Evol..

[37] Goerke C., Wirtz C., Fluckiger U., Wolz C. (2006). Extensive phage dynamics in *Staphylococcus aureus* contributes to adaptation to the human host during infection. Mol. Microbiol..

[38] Duquette R.A., Nuttall T.J. (2004). Methicillin-resistant *Staphylococcus aureus* in dogs and cats: An emerging problem?. J. Small Anim. Pract..

[39] Weese J., Dick H., Willey B., McGeer A., Kreiswirth B., Innis B., Low D. (2006). Suspected transmission of methicillin-resistant *Staphylococcus aureus* between domestic pets and humans in veterinary clinics and in the household. Vet. Microbiol..

[40] van Duijkeren E., Moleman M., van Oldruitenborgh-Oosterbaan M.S., Multem J., Troelstra A., Fluit A., van Wamel W., Houwers D., de Neeling A., Wagenaar J. (2010). Methicillin-resistant *Staphylococcus aureus* in horses and horse personnel: An investigation of several outbreaks. Vet. Microbiol..

[41] Choo E.J. (2017). Community-associated methicillin-resistant *Staphylococcus aureus* in nosocomial infections. Infect. Chemother..

[42] Kouyos R., Klein E., Grenfell B. (2013). Hospital-community interactions foster coexistence between methicillin-resistant strains of *Staphylococcus aureus*. PLOS Pathog..

[43] Otto M. (2012). MRSA virulence and spread. Cell. Microbiol..

[44] Loeffler A., McCarthy A., Lloyd D.H., Musilová E., Pfeiffer D.U., Lindsay J.A. (2013). Whole-genome comparison of meticillin-resistant *Staphylococcus aureus* CC22 SCC*mec*IV from people and their in-contact pets. Vet. Dermatol..

[45] Strommenger B., Kehrenberg C., Kettlitz C., Cuny C., Verspohl J., Witte W., Schwarz S. (2005). Molecular characterization of methicillin-resistant *Staphylococcus aureus* strains from pet animals and their relationship to human isolates. J. Antimicrob. Chemother..

[46] Vincze S., Brandenburg A.G., Espelage W., Stamm I., Wieler L.H., Kopp P.A., Lübke-Becker A., Walther B. (2014). Risk factors for MRSA infection in companion animals: Results from a case–control study within Germany. Int. J. Med. Microbiol..

[47] Harrison E.M., Weinert L.A., Holden M.T.G., Welch J.J., Wilson K., Morgan F.J.E., Harris S.R., Loeffler A., Boag A.K., Peacock S.J. (2014). A shared population of epidemic methicillin-resistant *Staphylococcus aureus* 15 circulates in humans and companion animals. mBio.

[48] Clinical and Laboratory Standards Institute—CLSI (2010). Performance Standards for Antimicrobial Susceptibility Testing, CLSI Supplement M100.

[49] Community Reference Laboratory for Antimicrobial Resistance—CRL—AR (2009). Multiplex PCR for the Detection of the mecA Gene and the Identification of Staphylococcus aureus.

[50] Stegger M., Andersen P., Kearns A., Pichon B., Holmes M., Edwards G., Laurent F., Teale C., Skov R., Larsen A. (2012). Rapid detection, differentiation and typing of methicillin-resistant *Staphylococcus aureus* harbouring either *mecA* or the new *mecA* homologue mecALGA251. Clin. Microbiol. Infect..

[51] Kaya H., Hasman H., Larsen J., Stegger M., Johannesen T.B., Allesøe R.L., Lemvigh C.K., Aarestrup F.M., Lund O., Larsen A.R. (2018). SCC*mec*Finder, a web-based tool for typing of staphylococcal cassette chromosome *mec* in *Staphylococcus aureus* using whole-genome sequence data. mSphere.

[52] Zankari E., Hasman H., Cosentino S., Vestergaard M., Rasmussen S., Lund O., Aarestrup F.M., Larsen M.V. (2012). Identification of acquired antimicrobial resistance genes. J. Antimicrob. Chemother..

